# Effect of speech volume on respiratory emission of oral bacteria as a potential indicator of pathogen transmissibility riska)

**DOI:** 10.1121/10.0002278

**Published:** 2020-10-23

**Authors:** Riyakumari K. Patel, Isis A. Shackelford, Mariah C. Priddy, Jonathan A. Kopechek

**Affiliations:** Department of Bioengineering, University of Louisville, Louisville, Kentucky 40292, USA

## Abstract

Respiratory droplets emitted during speech can transmit oral bacteria and infectious viruses to others, including COVID-19. Loud speech can generate significantly higher numbers of potentially infectious respiratory droplets. This study assessed the effect of speech volume on respiratory emission of oral bacteria as an indicator of potential pathogen transmission risk. Loud speech (average 83 dBA, peak 94 dBA) caused significantly higher emission of oral bacteria (*p* = 0.004 compared to no speech) within 1 ft from the speaker. N99 respirators and simple cloth masks both significantly reduced emission of oral bacteria. This study demonstrates that loud speech without face coverings increases emission of respiratory droplets that carry oral bacteria and may also carry other pathogens such as COVID-19.

## I. INTRODUCTION

The emergence of a new coronavirus, SARS-CoV-2, has caused major public health and economic impacts. SARS-CoV-2 can cause COVID-19 disease, with severe symptoms including respiratory distress and pneumonia (Zheng, 2020). Transmission of viral particles via respiratory droplets has been identified as a primary mode of infection (Zhang *et al.*, 2020). Further studies are needed to better understand the impact of speech volume and other factors on emission of potentially infectious respiratory droplets.

Respiratory droplets are known to carry a variety of pathogens, including influenza flu virus, *Mycobacterium tuberculosis* bacteria, which can cause tuberculosis, *Neisseria meningitidis* bacteria, which can cause meningitis, and SARS-CoV-2 found in both symptomatic and asymptomatic patients of COVID-19 (Tzeng *et al.*, 2014; Ma *et al.*, 2020; Stadnytskyi *et al.*, 2020). Recent studies found that loud speech can generate thousands of respiratory droplets per minute, which can increase the risk of COVID-19 transmission (Stadnytskyi *et al.*, 2020). These droplets can range in size from small aerosol droplets with diameters less than 5 *μ*m, which can remain in the air for hours, to larger respiratory droplets up to 100 *μ*m in diameter, which settle within minutes (Chao *et al.*, 2009; Stilianakis and Drossinos, 2010; Asadi *et al.*, 2019). Although COVID-19 viral particles are generally less than 140 nm in diameter, transmission often occurs through respiratory droplets, which can be >5–10 *μ*m in diameter (Stadnytskyi *et al.*, 2020). For comparison, bacteria are typically larger than 1 *μ*m in diameter but can also be transmitted via respiratory droplets with diameters >5–10 *μ*m (Welch *et al.*, 2016).

Prior studies have found that elevated vocalization increases the number of respiratory droplets emitted. For example, singing was shown to produce six times more respiratory droplets than speaking (Loudon and Roberts, 1968). Elevated speech volume has also been shown to increase emission of respiratory droplets (Asadi *et al.*, 2019). However, a quantitative comparison of speech volume with respiratory emission of oral bacteria, as an indicator of potential transmission of pathogens such as COVID-19, has not been previously described. Furthermore, the impact of masks on respiratory emission of oral bacteria during loud speech has not been quantified. Therefore, the objective of this study was to evaluate the effect of speech volume on emission of oral bacteria, which could yield new insights regarding the potential role that loud speech may have on increasing the risk of pathogen transmission.

## II. METHODS

### A. Experimental procedures

Respiratory emission of oral bacteria during speech was evaluated by culturing samples with two techniques for comparison: (1) agar plate culture and (2) liquid culture. Samples for agar plate culture were collected using 100-mm petri dishes filled with 20 mL of autoclaved LB agar gel. Samples for liquid culture were collected using 35-mm petri dishes filled with 5 mL of autoclaved Miller's LB broth (VWR, Radnor, PA).

**FIG. 1.**
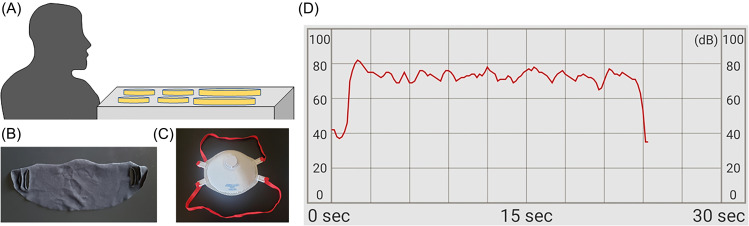
(Color online) (A) Schematic of the experiment setup for detection of respiratory oral bacteria emitted during speech. Samples were collected in large (100-mm) petri dishes for agar plate culture and in small (35-mm) petri dishes for liquid culture. Photos of (B) a simple cloth mask and (C) an N99 respirator mask used to assess the effect of face masks on emission of respiratory oral bacteria during speech. (D) Representative time trace of recorded sound pressure level during reading of the Rainbow Passage (unweighted sound pressure level during speech, which corresponds to average A-weighted value of 83 dBA and peak of 94 dBA).

To perform the experiment, agar plate and liquid culture samples were placed at a height of approximately 0.5 ft below the speaker [Fig. 1(A)], unless otherwise indicated. This distance was chosen to allow exposure of all agar plates and liquid culture samples in the experiment. The speaker read the Rainbow Passage out loud for approximately 20 s, which is a phonemically balanced text frequently used in respirator fit testing (Schwartz *et al.*, 2018). Sound pressure levels were measured at a distance of 1 ft from the speaker using the Sound Meter app (Smart Tools Co., Daegu, South Korea) on a Galaxy S9 (Model Number SM-G960U, Samsung, Seoul, South Korea) running Android 10, similar to previously described methods (Kardous and Shaw, 2014). A-weighted sound pressure levels were calibrated using a sound pressure level meter (BAFX Products, Scottsdale, AZ). The bandwidth of the microphone was 20 Hz–22 kHz. The recorded ambient noise level in the room was 32 dBA without speech. The experiments were conducted in a laboratory which meets minimum standards outlined in the National Institutes of Health (NIH) design requirements manual. Specifically, room temperature was maintained between 72 and 74 °C, relative humidity was between 45% and 55%, and there was a minimum of 12 air changes per hour.

Experiments were conducted with speech recorded at average sound pressure levels of 63 dBA (peak 75 dBA), 69 dBA (peak 77 dBA), 76 dBA (peak 86 dBA), 83 dBA (peak 94 dBA). In addition, experiments were also conducted with speech at average sound pressure levels of 84 dBA (peak 94 dBA) while wearing a simple single-ply cotton cloth mask, and 89 dBA (peak 96 dBA) while wearing an N99 respirator mask [Figs. 1(B) and 1(C)]. There was one speaker for all experiments in this study. The speaker drank water between each experiment to maintain similar hydration for each speech test. The speaker continuously monitored the sound pressure level during speech in order to maintain the average sound pressure level for each sample group. The amount of vocal effort required for average sound pressure levels above 77 dBA was similar with masks and without masks. Additional experiments tested speech at an average sound pressure level of 83 dBA (peak 94 dBA) with samples placed at distances of 0.5, 1, 2, 4, and 6 ft from the speaker. A representative time trace of the recorded sound pressure level during loud speech (average 83 dBA, peak 94 dBA) is shown in Fig. 1(D). Saliva samples (1 mL) were used as positive controls. Samples with no speech (negative control) were exposed to room air for 30 s while the speaker was sitting silently at a distance of 0.5 ft from the samples.

After the speech experiment was conducted, agar plates were incubated for 48 h at 37 °C in a lab oven, and liquid culture samples were transferred to plastic 100-mm culture tubes and incubated for 24 h at 37 °C in a shaking incubator at a speed setting of 200 (Gyromax 767, Amerex Instruments, Inc., Lafayette, CA).

### B. Data acquisition and analysis

Bacteria levels after liquid culture were determined by adding 100 *μ*L aliquots of each sample into a 96-well plate and measuring optical density at 660 nm (OD_660 nm_) as previously described (Brochu *et al.*, 1998; Koistinen *et al.*, 2007) using a platereader (BioTek, Winooski, VT). Photos of agar plate cultures were analyzed with NIH ImageJ software to calculate the % filled area within a 75-mm diameter circular region of interest (ROI) placed at the center of each 100-mm diameter petri dish. The outer rim of the petri dish was excluded from the ROI to avoid optical artifacts caused by the edges of the dish which would affect the analysis of filled area. The green channel was converted to a binary image by measuring the modal pixel intensity and adding 30 pixel intensity units to determine the threshold value. The addition of 30 pixel intensity units to the threshold value was used to eliminate noise from the image during conversion to a binary image. The percentage of area filled with bacteria colonies was computed within the ROI.

### C. Statistical analysis

Statistical analysis was performed using minitab (State College, PA). A *p*-value below 0.05 was considered statistically significant. One-way analysis of variance (ANOVA) was used for comparisons between multiple groups with *post hoc* analysis using Tukey's test. Mann-Whitney U tests were used for comparisons between two groups. A Bonferroni correction was used when performing multiple Mann-Whitney U tests on the same data set. Results are plotted as mean ± standard error of the mean (SEM).

## III. RESULTS

**FIG. 2.**
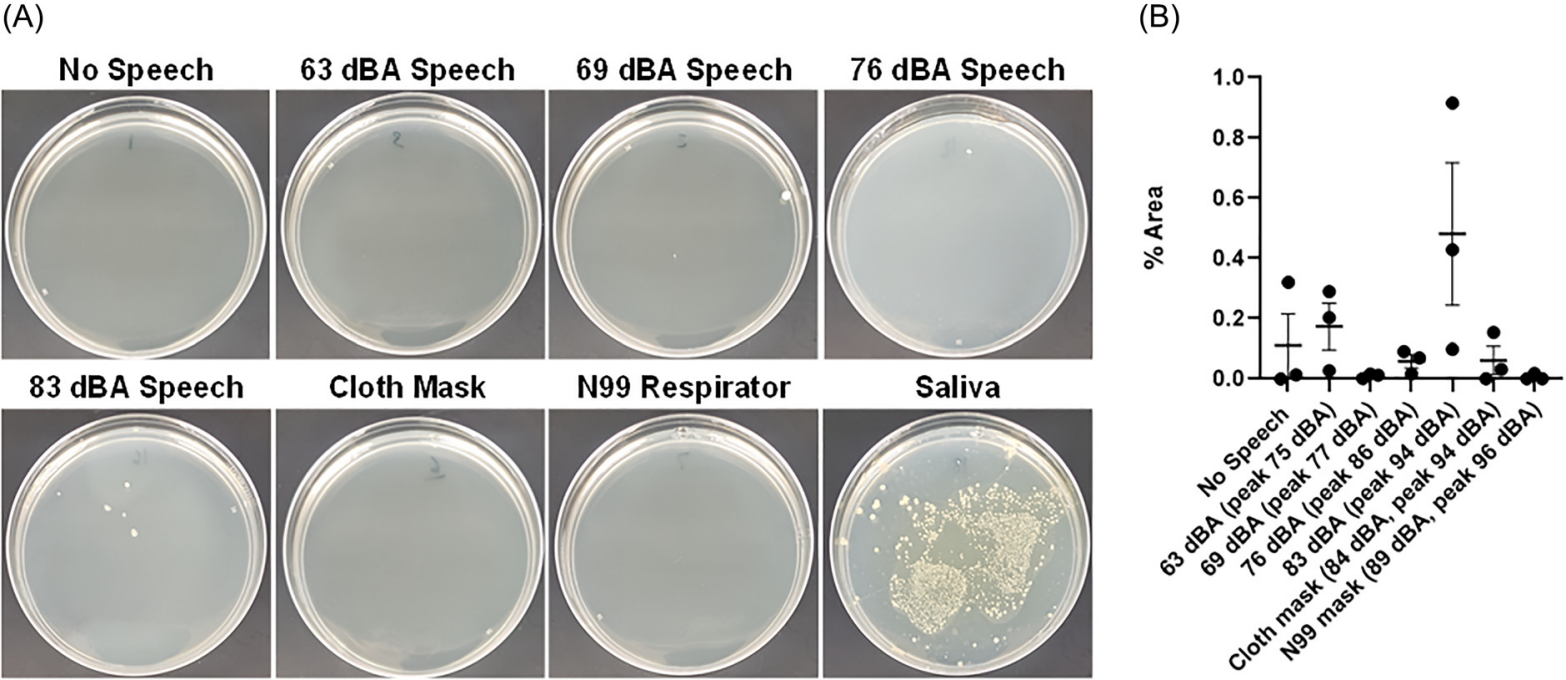
(Color online) (A) Representative images of agar plates 48 h after exposure to various conditions, including different levels of speech volume with and without face masks. White spots indicate growth of oral bacteria colonies that may be emitted via respiratory droplets during speech. (B) Analysis of agar plates indicates that loud speech volumes may increase the amount of oral bacteria that are emitted via respiratory droplets, which can carry pathogens such as COVID-19 (ANOVA *p* = 0.03). *Post hoc* analysis indicated that the 83 dBA speech group was statistically different from the no speech group, the 69 dBA group, and the N99 mask group.

Experimental results indicated that speech volume can affect respiratory emission of oral bacteria via respiratory droplets, which can also carry COVID-19 and other pathogens. Agar plates were cultured for 48 h to visually assess growth of bacteria colonies after exposure to various conditions. As shown in Fig. 2(A), bacteria colonies were evident in agar plates 48 h after exposure to speech at average sound pressure levels between 69 and 83 dBA, with the highest number of colonies observed at the highest speech volume tested (83 dBA average sound pressure level). No bacteria colonies were observed when the speaker wore a simple cloth mask or a more advanced N99 respirator mask, or in the negative control group (no speech). As expected, many colonies grew in the positive control group (saliva sample). Images were analyzed to determine the percentage of plate area that was covered by bacteria colonies. Samples exposed to saliva grew colonies on 14 ± 3% of the agar plate (*n* = 3), but all speech volume conditions had colonies covering less than 1% of the plate as shown in Fig. 2(B). Excluding saliva samples, there was a statistically significant difference in filled area between the other groups (ANOVA *p* = 0.035, *n* = 3/group). *Post hoc* analysis indicated that the 83 dBA speech group was statistically different from the no speech group (*p* = 0.043), the 69 dBA group (*p* = 0.047), and the N99 mask group (*p* = 0.044). There was a bacteria colony in one sample without speech, which may have been caused by bacteria contamination from the air, but this control group was still statistically different compared to the 83 dBA speech group. It is important to note that the oral bacteria grow at a logarithmic rate and each bacterium emitted during speech can replicate into thousands of new bacteria after 24 or 48 h of culture. This can cause large differences in the measured bacteria levels even if initial differences in levels of emitted bacteria were low, but in this study statistically significant differences in the measured bacteria levels were still observed despite the inherent variability due to logarithmic growth. Overall, these results suggest that the amount of respiratory droplets emitted during speech depends on the speech volume.

**FIG. 3.**
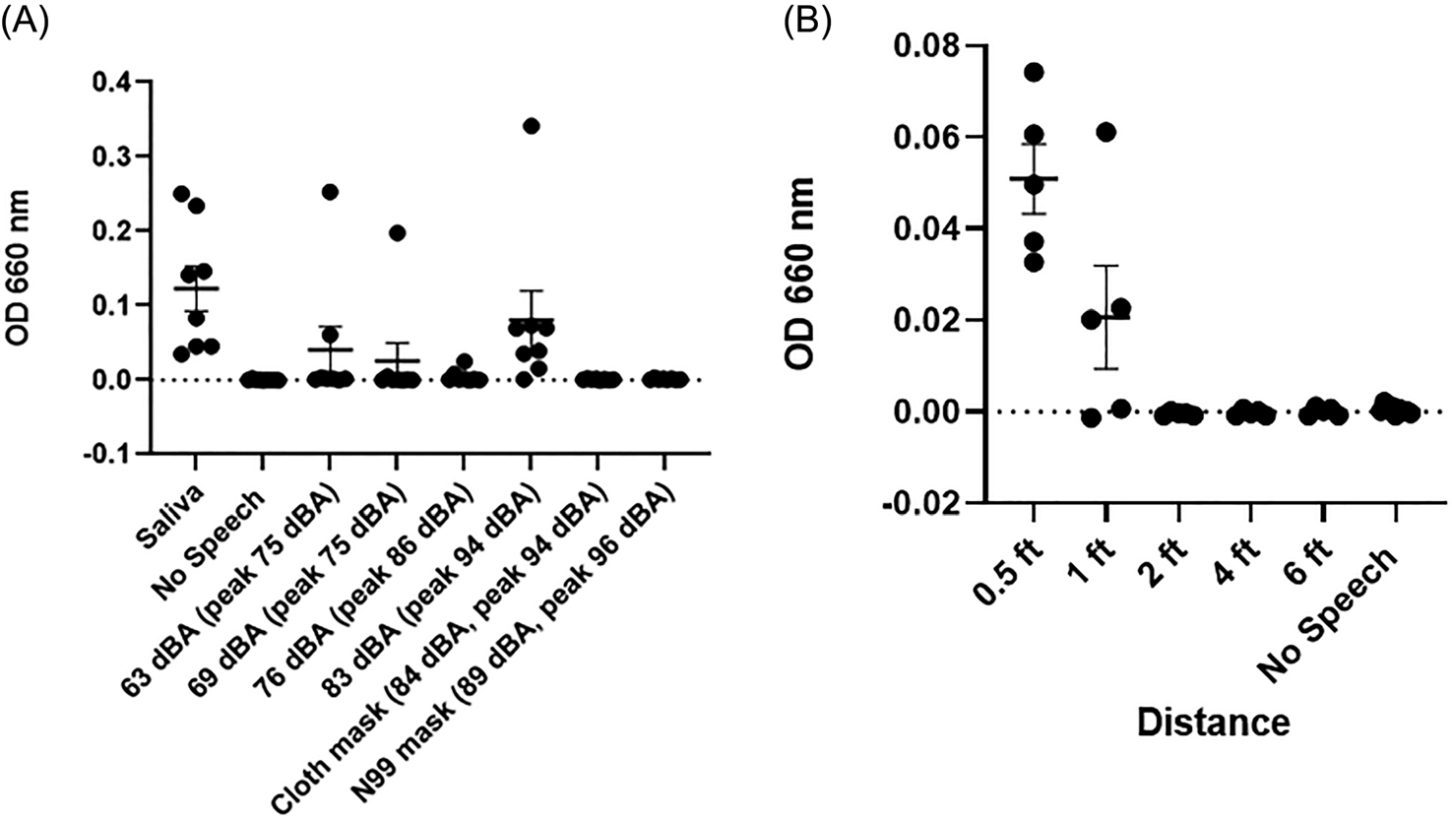
(A) Optical density measurements (as an indicator of bacteria concentration) in liquid culture samples exposed to various conditions. There was a significant increase (*p* < 0.05) in bacteria levels for saliva (positive control) and the loudest speech volume (83 dBA), but no significant difference (*p* > 0.05) was observed when the speaker wore either a cloth mask or an N99 respirator mask (*n* = 8–9/group). (B) At the loudest speech volume (83 dBA), bacteria levels were significantly increased at a distance of 0.5 ft from the speaker but not at further distances (*n* = 5/group).

Quantitative photometric assessment of bacteria in liquid culture indicated that higher speech volume caused increased emission of oral bacteria via respiratory droplets. As shown in Fig. 3(A), there was a significant increase in optical density (indicating increased bacteria concentration) for both the saliva samples and the loudest speech volume (83 dBA) compared to the no speech condition (*p* < 0.05, *n* = 8–9/group). In addition, there was a statistically significant difference in optical density between the loudest speech volume (83 dBA) and both conditions where the speaker wore a mask (*p* < 0.05 for both cloth mask and N99 respirator groups). No statistically significant difference in optical density was observed between the no speech group and groups where the speaker wore a mask (*p* > 0.05). These results indicate that loud speech volumes can cause emission of oral bacteria via respiratory droplets, but this was significantly reduced by wearing an N99 respirator mask or even a simple cloth mask.

Additional experiments were performed to assess the level of oral bacteria emitted at various distances from the speaker using the loudest speech volumes tested (83 dBA) without wearing a mask. As shown in Fig. 3(B), there was a significant increase in optical density at a distance of 0.5 ft from the speaker, but bacteria levels decreased at further distances from the speaker. These results demonstrate that physical distance affects the level of exposure to respiratory droplets which can contain oral bacteria or other pathogens.

## IV. DISCUSSION

The results of this study provide insights into the effect of speech volume on respiratory emission of oral bacteria via respiratory droplets that can carry infectious particles, such as COVID-19. Oral bacteria emission occurred at all speech levels tested in this study (average sound pressures levels of 63–83 dBA) and there was some variability in the results at lower speech volumes, but significantly higher levels of oral bacteria emission occurred at the loudest speech level (83 dBA). It is well established that loud speech, such as shouting or singing, emits significantly higher numbers of respiratory droplets and can increase the risk of airborne pathogen transmission (Bourouiba, 2020; Stadnytskyi *et al.*, 2020). However, to our knowledge this is the first study to directly compare measurements of speech volume with the level of oral bacteria emitted as an indicator of respiratory droplet emission. Furthermore, this study indicates that wearing masks, including a simple cloth mask, can significantly reduce respiratory emission of oral bacteria during loud speech. There is evidence that homemade masks used widely by the public can reduce transmission of pathogens such as COVID-19 by filtering larger respiratory droplets (Eikenberry *et al.*, 2020). The levels of oral bacteria emission were slightly higher with cloth masks compared to N99 respirator masks but the differences between each type of mask were not statistically significant. Only N95 or N99 respirator masks are certified to filter pathogens from aerosols, and other face coverings such as cloth masks or surgical masks can differ in effectiveness based on their permeability and material properties. However, all types of face coverings can have some effect on reducing emission of respiratory droplets that could potentially spread pathogens to others in the vicinity. It should be noted that these results do not provide any insights into whether cloth masks can help protect the wearer, but they do provide additional evidence that wearing masks can potentially reduce pathogen transmission to other people nearby, consistent with other recent studies (Lyu and Wehby, 2020; Zhang *et al.*, 2020).

There were several factors that should be considered when comparing the results of this study to potential risks of pathogen transmission in other settings. The Rainbow Passage that was read out loud for these experiments was less than 30 s in duration. Longer speech durations would significantly increase the level of respiratory droplet emission. Sustained loud speech above 83 dBA, which can sometimes occur in loud restaurants/bars, sporting events, and similar settings, would likely emit much higher levels of respiratory droplets and significantly increase the risk of COVID-19 transmission (Asadi *et al.*, 2019). Loud singing would also be expected to generate high levels of respiratory droplets.

This study found lower levels of oral bacteria emission at lower speech volumes. In addition, emitted oral bacteria levels were very low beyond 1 ft from the speaker. The distance of respiratory droplets can be affected by multiple factors including air flow, temperature, humidity, droplet size, etc., so it is possible that oral emission of pathogens, such as COVID-19, could extend beyond 1 ft from the speaker in other conditions. Furthermore, recent evidence indicates that COVID-19 may be transmitted via aerosol droplets that can remain suspended in the air for hours and travel well beyond 6 ft (Fears *et al.*, 2020). In addition, emission rates of respiratory droplets can vary significantly between different individuals even at the same speech volume. Activities that increase saliva production, such as eating and drinking, can also affect emission rates of respiratory droplets and oral bacteria or other pathogens. This study was performed with a speaker lacking any symptoms of COVID-19 and presumed to be non-infected, but it is currently unknown whether COVID-19 infection can affect the emission rates of respiratory droplets due to possible differences in saliva surface tension or viscosity. Regardless, these results clearly demonstrate that quieter speech and increased distance from the speaker can decrease respiratory droplet exposure and reduce the risk of pathogen inhalation.

This study did not directly measure respiratory emission of COVID-19 virus or other pathogens during speech due to the significant challenges associated with safely collecting and handling those samples in this type of study. However, there is clear evidence that both oral bacteria and infectious pathogens, including COVID-19, can be transmitted via a similar process involving respiratory droplets (Loudon and Roberts, 1968; Fears *et al.*, 2020; Zhang *et al.*, 2020). Therefore, the results of this study provide new insights into the potential impact of speech volume on the risk of transmission of pathogens, such as COVID-19, via respiratory droplets.

## V. CONCLUSION

This study demonstrates that higher speech volumes increase the levels of respiratory droplets emitted that contain oral bacteria and can also transmit pathogens such as COVID-19. Respiratory emission of oral bacteria was reduced by decreasing speech volume or by increasing distance from the speaker. Wearing an N99 respirator mask or even a simple cloth mask significantly reduced emission of oral bacteria during loud speech. These results provide new evidence that quieter speech and wearing masks can potentially reduce the risk of transmitting pathogens such as COVID-19.
